# Pyonephrosis Ultrasound and Computed Tomography Features: A Pictorial Review

**DOI:** 10.3390/diagnostics11020331

**Published:** 2021-02-17

**Authors:** Stefania Tamburrini, Marina Lugarà, Michele Iannuzzi, Edoardo Cesaro, Fiore De Simone, Dario Del Biondo, Roberta Toto, Dora Iulia, Valeria Marrone, Pierluigi Faella, Carlo Liguori, Ines Marano

**Affiliations:** 1 Department of Radiology, Ospedale del Mare ASL NA1 Centro, 80147 Naples, Italy; desimonef@libero.it (F.D.S.); valeriamarrone@hotmail.it (V.M.); pierluigifaella@libero.it (P.F.); carlo.liguori@gmail.com (C.L.); ines.marano@tiscali.it (I.M.); 2 Department of Internal Medicine, Ospedale del Mare ASL NA1 Centro, 80147 Naples, Italy; marinalugara82@gmail.com; 3 Department of Anesthesia and Critical Care, Ospedale del Mare ASL NA1 Centro, 80147 Naples, Italy; michele.iannuzzi74@gmail.com (M.I.); rtoto@libero.it (R.T.); 4 Department of Radiology, Università degli Studi Della Campania Luigi Vanvitelli, 80138 Naples, Italy; edoardo.cesaro@yahoo.it; 5 Department of Urology, Ospedale del Mare ASL NA1 Centro, 80147 Naples, Italy; dariodelbiondo@gmail.com; 6 Department of Clinical Pathology, Ospedale del Mare ASL NA1 Centro, 80147 Naples, Italy; dora.iulia@gmail.com

**Keywords:** urinary tract infections, CT, US, pyonephrosis, sepsis

## Abstract

Urinary tract infections (UTIs) are the most frequent community-acquired and healthcare-associated bacterial infections. UTIs are heterogeneous and range from rather benign, uncomplicated infections to complicated UTIs (cUTIs), pyelonephritis and severe urosepsis, depending mostly on the host response. Ultrasound and computed tomography represent the imaging processes of choice in the diagnosis and staging of the pathology in emergency settings. The aim of this study is to describe the common ultrasound (US) and computed tomography (CT) features of pyonephrosis. US can make the diagnosis, demonstrating echogenic debris, fluid/fluid levels, and air in the collecting system. Although the diagnosis appears to be easily made with US, CT is necessary in non-diagnostic US examinations to confirm the diagnosis, to demonstrate the cause and moreover to stage the pathology, defining extrarenal complications. In emergency settings, US and CT are differently used in the diagnosis and staging of pyonephrosis.

## 1. Introduction

Urinary tract infections (UTIs) are amongst the most frequent community-acquired and healthcare-associated bacterial infections, and they are to be found in many specialties, such as internal medicine, gynecology, urology and intensive care medicine. UTIs are classified based on the anatomical level of infection, the severity grade of infection, underlying risk factors and microbiological findings. However, the clinical phenotypes of UTIs are heterogeneous and range from benign, uncomplicated infections to complicated UTIs (cUTIs), pyelonephritis and severe urosepsis, depending mostly on the host response. Pyonephrosis (PN) refers to infected hydronephrosis with associated suppurative destruction of the kidney parenchyma, with partial or total loss of renal function, and it is defined as an accumulation of purulent debris and sediment in the renal pelvis and urinary collecting system. PN is associated with factors that compromise host defense, including immunosuppression, renal failure, renal transplantation [1,2], and it is associated with local factors that compromise the urinary tract, such as congenital uropathy, neurogenic bladder, pregnancy, presence of foreign bodies such as calculi, indwelling catheters or other drainage devices, or endoscopic maneuvers, and it is frequently associated with the obstruction of the collecting system (renal calculus disease, RCD) [2,3,4,5,6]. PN has also been reported in patients with urinary obstruction from tumors, such as in transitional cell carcinoma [7,8]. Emerging evidence challenges the current paradigm that the bladder is a sterile microenvironment, and reveals that live bacteria are present in the bladder, even in “culture negative” patients [9,10,11,12]. Under basal conditions, the microbiome is not simply an innocent bystander, living peacefully side-by-side with its human host. Rather, commensal microbes are health-promoting and play numerous diverse roles in the maintenance of human wellness. In contrast, the microbiome is altered in multiple disease states with the conversion of the health-inducing microbiome into a disease-promoting microbiome (also known as pathobiome). This condition is particularly pronounced in critically ill patients, where the pathobiome plays an important role in critically ill infection [13,14,15].

Pyonephrosis is uncommon in adults, rare in children and extremely rare in neonates; however, it has been reported in several neonates and adults, making it clear that the condition may develop in any age group. The clinical spectrum of PN is extremely variable, from asymptomatic bacteriuria to sepsis, making diagnosis more challenging. Similar to an abscess, pyonephrosis is typically associated with fever, chills and flank pain, although some patients may be asymptomatic. The walled-off exudate is protected by the body’s natural system and by antibiotics, and for those reasons a prompt diagnosis at an early stage is necessary, because the systemic inflammatory response syndrome leading to major organ dysfunction (fever or hypothermia, hyperleukocytosis or leukopenia, tachycardia, hypotension, tachypnea, neurological compromise (q-SOFA)) is recognized as the first event in a cascade to multi-organ failure. For these reasons, PN is considered a urological emergency that can rapidly progress to sepsis and septic shock [6,16]. The treatment of urosepsis calls for the combination of urgent decompression (either percutaneous or retrograde with a ureteral stent placement under US or CT guidance), adequate life-supporting care, appropriate and prompt antibiotic therapy, and adjunctive measures. Drainage has low morbidity and mortality rates and an excellent outcome, and significantly decreases the need for nephrectomy. The prognosis of pyonephrosis is good in patients who receive prompt diagnosis and treatment, with the infectious process rapidly resolving after either nephrostomy or retrograde stent drainage, because of the adequate control of overwhelming infection in an obstructed renal unit [3,17,18,19,20]. When pyonephrosis is not recognized early, it can rapidly deteriorate and develop into septic shock, and in these cases potential complications include irreversible damage to the kidneys with the possible need for nephrectomy [21]. Ultrasound and computed tomography represent the imaging of choice in the diagnosis and staging of the pathology in emergency settings. In clinical practice the distinction between PN and hydronephrosis is challenging.

Ultrasound is easily performed in emergency settings and bedside for the critically ill patients, and is often used in image guidance for interventions and to monitor the drainage of the calico-pelvic system [3,20,22,23]. Ultrasound can rapidly confirm or exclude the presence of pyonephrosis, speeding up patient management and treatment, and demonstrate hydronephrosis with echogenic debris in the renal pelvis, which is considered highly suggestive of pyonephrosis [18,24,25,26]. Although computed tomography (CT) is considered more reliable, useful, and impactful in the critical care setting [22,23,27,28,29,30,31], the diagnosis can be missed on unenhanced CT because of the difficulty encountered in the radiologic differentiation of hydronephosis from pyonephrosis [31]. The direct assessment of purulent urine is easily performed in the case of the presence of air bubbles; recent studies demonstrated the HU measurement of urine may be helpful in the diagnosis of purulent urine [27,29,30,31]. At contrast-enhanced CT examinations, PN signs are characterized by the presence of signs of obstruction with associated renal pelvic wall thickening and perirenal fat stranding [27,28,32,33]. This pictorial review will highlight the common US and CT features of pyonephrosis in adults that may help to rapidly diagnose and stage pyonephrosis.

## 2. MeSH Terms

Terms collected from Pubmed (National Center for Biotechnology Information, 8600 Rockville PikeBethesda, MD 20894) in December 2020, including review studies, scientific papesr, case report, case series.

- adult;
- contrast media;
- diagnosis, differential;
- female/male;
- humans;
- hydronephrosis/diagnosis;
- image enhancement/methods;
- kidney/pathology;
- pyelonephritis/diagnosis;
- reproducibility of results;
- sensitivity and specificity;
- genito urinary disease;
- kidney disease;
- genito urinary infection complications;
- hydronephrosis, infected;
- infected hydronephrosis;
- male urogenital diseases;
- urologic diseases;
- kidney diseases;
- hydronephrosis;
- female urogenital diseases and pregnancy complications;
- female urogenital diseases;
- urologic diseases;
- kidney diseases;
- hydronephrosis

## 3. Ultrasound

**Figure 1:**
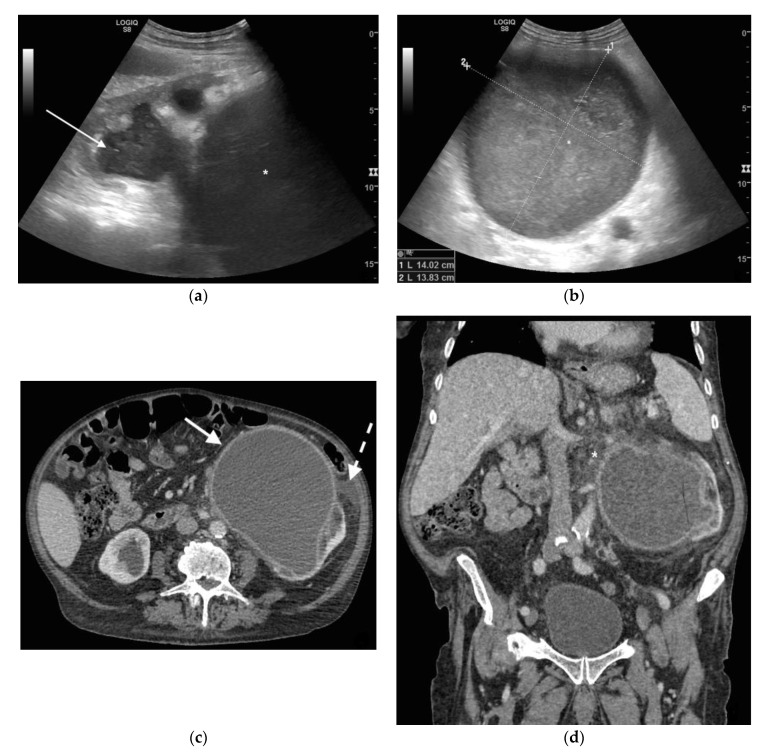
A 78 years old septic male patient. Ultrasound (US) longitudinal (**a**) and axial (**b**) view of the left kidney showing high-grade hydronephrosis with gross dilatation of the renal pelvis and calyces, filled by inhomogeneous urine (white arrow) and an extremely dilated inferior calyceal group filled by a huge ball of debris conglomerate (*). Axial (**c**) and MPR coronal Computed Tomography (CT) (**d**) after intravenous contrast in cortical-medullary phase, showing high grade hydronephrosis, diffuse parietal thickening of the calico-pelvic system (white arrow), fat stranding of perirenal and renal sinus fat. Small amount of free fluid is appreciated anteriorly (dashed arrow).

**Figure 2:**
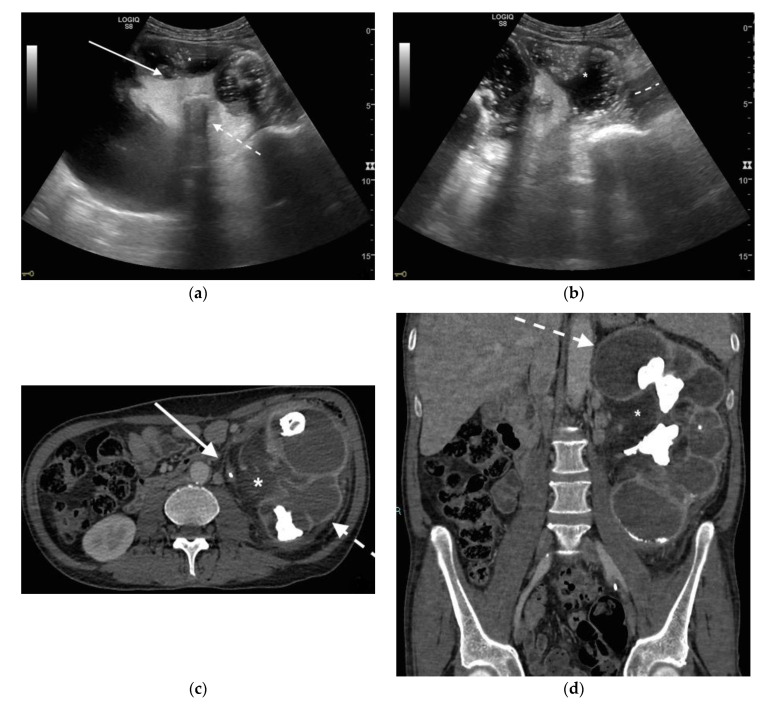
A 71 years old septic patient. US longitudinal (**a**,**b**) view of left kidney. Severe hydronephrosis, with extreme cortical thinning. High-grade dilatation and parietal thickening (white arrow) of calico-pelvic system fluid filled by inhomogeneous fluid with multiple hyperechogenic spots (*). Enlarged and markedly hyperechogenicity of renal sinus fat with acoustic shadowing due to staghorn calculi (dashed arrow). Perirenal fat inhomogeneity was detected (dashed line). Axial (**c**) and MPR coronal (**d**) CT with intravenous contrast in cortical-medullary phase after stent placement (white arrow), showing high-grade hydronephrosis with diffuse parietal thickening of calico-pelvic system (dashed arrow), staghorn calculi and renal sinus lipomatosis with mild inhomogeneous fat stranding and suffusion (*).

**Figure 3:**
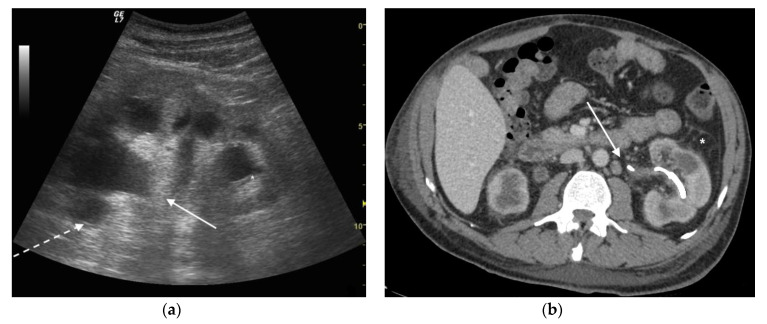
A 73 years old septic male patient. US longitudinal view of left kidney (**a**,**b**) showing hydronephrosis with parietal thickening of inferior calyceal group (*), dense peripheral echoes within the calyceal system (white arrow) with acoustic shadowing at low-gain indicating gas-forming infection, perirenal suffusion was detected (dashed arrow). Axial CT (**b**) with intravenous contrast in cortical-medullary phase after stent placement (white arrow), showing drainage of calico-pelvic system. Mild perirenal fat stranding (*).

**Figure 4:**
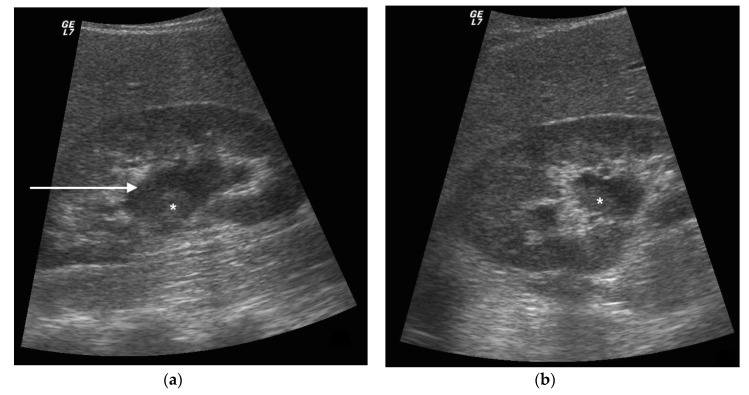
A 42 years old septic female patient. US longitudinal view (**a**,**b**) of the right kidney showing low/mild-grade hydronephrosis (white arrow) with sharply defined urine debris level (*).

**Figure 5:**
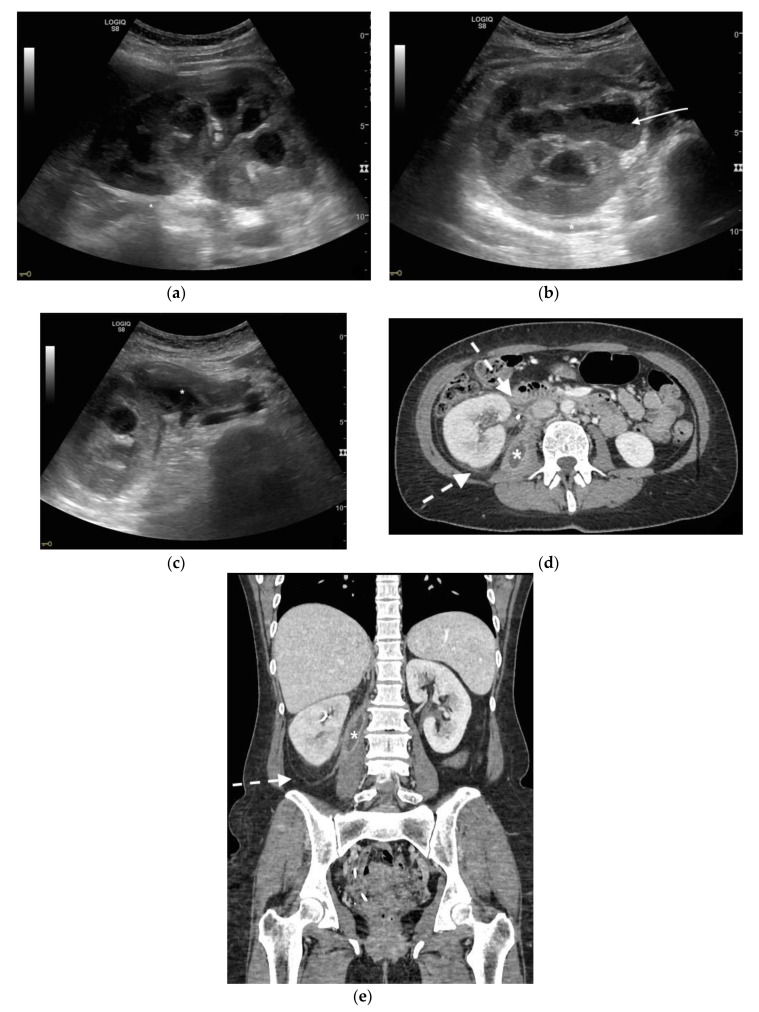
A 48 years old septic woman. US longitudinal (**a**) and axial (**b**,**c**) view of the right kidney. Enlargement of the kidney, hydronephrosis with sharply defined urine-debris level (white arrow). Thickening of perirenal fascia and markedly inhomogeneous echogenicity of perirenal fat suggesting extrarenal extension (*). The patient underwent nephrostomy in the emergency setting, and CT was performed after stent placement. Axial (**d**) and MPR coronal (**e**) CT with intravenous contrast in parenchymal phase, showing correct stent positioning (white arrow) and drainage of calyceal-pelvic system. The right kidney appeared enlarged with a delayed parenchymal phase, the calyceal-pelvic system was unstretched after nephrostomy placement. Right perirenal fascia was thickened posteriorly (dashed arrow) and extrarenal extension of the pathology was confirmed by the presence of an abscess in the right iliopsoas muscle (*).

**Figure 6:**
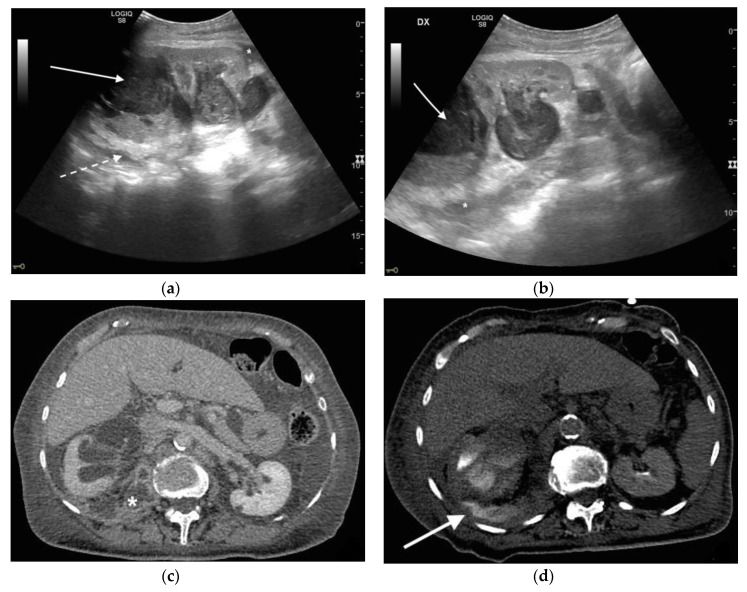
An 83 years old septic woman. US longitudinal (**a**,**b**) view of the right kidney showing high-grade hydronephrosis with the calyceal-pelvic system completely occupied by inhomogeneous echogenic debris (with arrow). Perirenal fascia is thickened and perirenal fat is hyperechogenic and inhomogeneous (dashed arrow). Fluid over-collection is appreciated around the kidney (*), and free fluid was appreciated in the abdomen. Complicated pyonephrosis with extrarenal extension was diagnosed. CT with intravenous contrast. Axial CT after intravenous contrast in parenchymal (**c**) and urography phase (**d**), showing hydronephrosis with parietal thickening of calyceal-pelvic system, multiple loculated over fluid collection in the perirenal (white arrow) space and abscess in iliopsoas muscle (*). In the urography phase, extravasation of urine into extrarenal fluid collections due to abscessualization of the calyceal-pelvic system.

Renal ultrasound has an established role in evaluating patients with suspected renal inflammatory disease [20,21,34]. Ultrasound is particularly effective in diagnosing pyonephrosis: the sensitivity of renal ultrasonography for differentiating hydronephrosis from pyonephrosis is 90%, and its specificity is 97% [21,24,34]. The key diagnostic point in differentiating urine from infected urine is that in the first case, the collecting system has the acoustic properties of a cystic structure; instead, ultrasonographic findings suggestive of pyonephrosis include the presence of hydronephrosis in conjunction with hyperechoic debris in the collecting system. These findings are specific enough: their absence excludes pyonephrosis with a high degree of accuracy (Figure 1 and Figure 2) [21,24,25,26,27,28,35]. In emphysematous pyelonephritis, the presence of gas in the US-B mode is observed as large hyperechoic lines appearing as “dirty shadows” (Figure 3) [36]. US can detect thickening of the renal pelvic wall (>2 mm), which in severe cases may appear multilayered due to mucosa edema (Figure 3). Ultrasonography does have drawbacks [37]; for example, it may not always differentiate hydronephrosis from early pyonephrosis, especially in the case of low-grade hydronephrosis (Figure 4), and the ultrasound diagnosis of PN can be challenging in patients with kidney abnormalities or in the presence of a parapyelic cyst. The major limit of ultrasound is represented by the extrarenal/retroperitoneal extension of the pathology, because of the intrinsic limit of the imaging modality, especially in obese patients. Extrarenal/retroperitoneal extension of pyonephrosis should be considered every time inhomogeneous fluid is seen around the kidney within the perirenal space, in the case of inhomogeneous hyperechogenicity of perirenal fat, or by the direct visualization of extrarenal abscess formation (Figure 5 and Figure 6). In those cases, second level imaging is mandatory to assist the clinician in planning renal decompression.

## 4. CT

CT in pyonephrosis is performed without or with intravenous contrast administration, depending on renal impairment. The presence of clinical signs of infection with hydronephrosis on CT is considered a more sensitive indicator of pyonephrosis than many CT findings alone.

CT signs of pyonephrosis are considered indirect; in fact, in addition to features of obstruction, CT may demonstrate thickening of the renal pelvic wall (>2 mm), parenchymal or perinephric inflammatory changes, dilatation and obstruction of the collecting system, and gas–fluid or fluid–fluid levels in the intrarenal collecting system [28,29,30,31]. A caveat of CT evaluation is that it is often difficult to distinguish simple hydronephrosis from pyonephrosis by fluid attenuation measurements, although recent studies have demonstrated that patients with pyonephrosis had higher Hounsfiled Units (HU) levels in the dilated collecting system than in the case of simple non-complicated hydronephrosis, supporting its utility in measuring the density of urine in those patients [29,30,31]. This may be especially important in patients that cannot undergo contrast-enhanced CT. In these patients, the evaluation of the HU of the obstructed collecting system may provide additional information to the clinician for a probable diagnosis of UTI. Using the HU in addition to the classical parameters (thickening of the renal pelvis and stranding of the perirenal fat) for pyonephrosis in CT-imaging allows responsible physicians to make earlier diagnoses and earlier interventions [30].

Generalized peritonitis can result from a rupture of the pyonephrotic kidney. Intraperitoneal and retroperitoneal spontaneous rupture has been quite frequently reported, making it possibly much more common than originally thought [33,37,38,39,40].

Fistula can develop and can be associated with peritonitis: renocolic, renoduodenal, renocutaneous are the most common [41,42]. Other rare complications reported are renal venous thrombosis, psoas abscess and/or perinephric abscess. CT stages pyonephrosis accurately, detecting extrarenal peritoneal and retroperitoneal complications. Locally, CT can clearly demonstrate renal and perinephric abscess that not infrequently involve the retroperitoneal spaces and ileopsoas muscle, and assess the presence of fistulae to pleura, colon and duodenum (Figure 5 and Figure 6).

CT staging is also extremely helpful to plan the approach for decompression.

## 5. Discussion

Hydronephrosis is the dilatation of the renal pelvis and calyces; on the other hand, pyonephrosis refers to infected hydronephrosis or “pus under pressure”, and it is considered a true medical emergency. Several studies have shown that the combination of obstructive uropathy and infection is the underlying cause of up to 85% of urosepsis [1,3]. Due to the high risk of renal function loss, sepsis, and sepsis-related morbidity and mortality, the rapid diagnosis and treatment of pyonephrosis are essential to avoid permanent loss of renal function and to prevent sepsis [16,17,18,19,43]. In clinical practice, the distinction between PN and uninfected hydronephrosis is challenging. Patients with pyonephrosis may present with signs and symptoms of acute infection, or with low-grade fever, weight loss and dull pain, although as many as 15% may be afebrile [1,3]. The aim of radiological exams in UTI is to detect conditions that should be corrected to avoid the imminent deterioration of kidney function or to prevent recurrent infections, to provide information about the nature and the extent of the disease, and to identify significant complications. Ultrasound (US) and computed tomography (CT) are the imaging methods of choice in emergency settings to evaluate patients with suspected complicated UTIs. Ultrasound and bedside ultrasound represent the first imaging modality with high diagnostic accuracy in critically ill patients [22,23]. Sonography is an accessible and cost-effective modality for evaluating renal infections, and the lack of ionizing radiation and iodinated contrast are additional benefits. US is a sensitive detector of the pelvicalyceal dilation of the collecting system that can be shown even in the absent function. US can detect stones on the bladder–ureter junction, but cannot easily show the normal ureter or ureteric calculi in the other position. When pyonephrosis is present, US is especially effective in demonstrating and differentiating hydronephrosis from pyonephrosis [24,27,44]. US findings include hydronephrosis with echogenic material in the collecting system, with a shifting urine debris level, with a 100% positive predictive value for suspected pyonephrosis [21,34,35]. With US, associated structures in the abdomen and pelvis can be imaged, which may reveal the cause or level of the obstruction. US accuracy may be limited in obese patient and in staging the retoperitoneal or peritoneal extension of the pathology; moreover, in acute obstruction, calyceal dilatation may be minimal and US can be misleading [22,23,24,34,35].

US has a limited diagnostic value in the case of emphysematous infections because the presence of the gas mimics renal calculi and the produced artifacts, due to the reverberation of echoes and shadows, so that the visualization of the kidney and perirenal liquid collection is labored and vague [36,44].

CT is the investigation of choice in adult hydronephrosis, as it depicts hydronephrosis and often its underlying cause. CT examination should be mandatory in non-diagnostic ultrasound exams and in staging the extrarenal extension of the pathology. However, CT diagnosis of pyonephrosis is mostly based on indirect signs, because the differentiation of simple hydronephrosis from pyonephrosis may be challenging on CT. The imaging of obstructions can be performed without the use of IV contrast material with an accuracy of 97% in the detection of ureteral calculi, although contrast-enhanced imaging is more desirable to determine infection parenchymal and functional changes. The CT indirect signs of the pyonephrosis of an infected collecting system in the setting of hydronephrosis are pelvic and ureteral wall thickness, renal enlargement, perinephric fat stranding and a striated nephrogram in the renal parenchyma, which is usually more severe in pyonephrosis than in obstructive uropathy. Pelvic wall thickening has a sensitivity of 76% for pyonephrosis, whereas a thickening of the fascia and bridging septa are suggestive but non-specific because they are frequently detected in association with other conditions (retroperitoneal neoplasms, inflammation, trauma, infarction, and peritonitis). Fluid–fluid levels and gas within the collecting system may occasionally be seen. Contrast layer inversion, representing contrast material overlying purulent fluid, is rarely encountered. Gas in the collecting system, in the absence of previous procedures, is the most accurate indicator of the presence of the infected fluid [28,29,30,33,34,44].

Boeri et al. [30] reported in a relatively large cohort of patients that patients with pyonephrosis have higher HU levels than those with simple pyonephrosis, demonstrating that HU determination had a good predictive ability in differentiating pyonephrosis from hydronephrosis; moreover, they demonstrated that HU values were higher in patients with sepsis. HU measurements (cut-off 7.3) could be used as a potential predictor for sepsis in patients with obstructive uropathy in the real-life setting. The pyonephrotic medium consists of infected material, cellular debris and microorganisms, all of which have the potential to increase the attenuation on a CT scan, and the rationale of using attenuation values in differentiating hydronephrosis from pyonephrosis relies on this phenomenon [31]. The efficacy of CT attenuation values in differentiating exudate from transudate has been reported in several studies [45,46]. Because of the lack of direct characterization of purulent urine via CT, the routine HU measurements of the obstructed collecting system should be performed to support the diagnosis and to increase diagnostic confidence [27,28,29,30,32,44].

US can accurately diagnose PN, demonstrating echogenic debris, fluid/fluid levels and air in the collecting system. Although the diagnosis appears to be easily made with US, CT is necessary in non-diagnostic US examination, to confirm the diagnosis, to demonstrate the cause, and moreover to stage the pathology defining extrarenal complications.

## 6. Conclusions

In emergency settings, US and computed tomography CT are the most frequent imaging examinations used to evaluate patients with suspected complicated UTIs. US is often the first imaging modality in emergency settings, based on its convenience, availability, lower cost, and high accuracy in cases of suspected pyonephrosis. CT is the most accurate modality for evaluating inflammatory renal parenchymal diseases, and in the case of pyonephrosis can confirm the diagnosis, detect the cause and stage the extrarenal extension of the disease process. US and CT findings are complementary and are usually combined to increase the final performance of the overall diagnostic assessment, and are both used as image guidance for interventions.

## Data Availability

The data presented in this study are available on request from the corresponding author.
